# ncRNA-Regulated LAYN Serves as a Prognostic Biomarker and Correlates with Immune Cell Infiltration in Hepatocellular Carcinoma: A Bioinformatics Analysis

**DOI:** 10.1155/2022/5357114

**Published:** 2022-11-08

**Authors:** Liangbin Cao, Lingling Zhu, Li Cheng

**Affiliations:** ^1^Department of Anaesthesiology, The First Affiliated Hospital of Wannan Medical College (Yijishan Hospital of Wannan Medical College), Wuhu, Anhui Province, China; ^2^NHC Key Laboratory of Hormones and Development, Tianjin Key Laboratory of Metabolic Diseases, Chu Hsien-I Memorial Hospital & Tianjin Institute of Endocrinology, Tianjin Medical University, Tianjin, China; ^3^Department of Urology, The First Affiliated Hospital of Wannan Medical College (Yijishan Hospital of Wannan Medical College), Wuhu, Anhui Province, China

## Abstract

Liver hepatocellular carcinoma (LIHC) remains a lethal disease for humans. Immune checkpoint inhibitors (ICIs) targeting PD1/PD-L1 and CTLA4 offered new hopes for advanced-stage patients. Novel immune biomarkers and therapeutic targets are urgently needed. For the first time, we evaluated the expression and prognostic value of Layilin (LAYN) using in silico analyses and uncovered the carcinogenic role of LAYN in LIHC. The HCG18/hsa-mir-148a/LAYN axis was predicted as the upstream mechanism. Moreover, gene set enrichment analysis (GSEA) revealed that LAYN and its coexpressed genes primarily participated in immune response pathways, and LAYN expression was found significantly correlated with tumor immune cell infiltration in LIHC tissues. In general, our data provided evidence that HCG18/hsa-mir-148a-regulated high expression of LAYN is associated with immune cell infiltration and unfavorable prognosis of LIHC patients.

## 1. Introduction

Liver hepatocellular carcinoma (LIHC) represents the vast majority of primary liver cancers, which leads to the second most cancer-related mortality worldwide [1]. Risk factors of developing LIHC include hepatitis B and C virus infection, alcohol addiction, fungal metabolite aflatoxin B1 intake, and newly proposed causes such as nonalcoholic fatty liver disease and metabolic maladies [2–4]. The long-term survival of LIHC patients remains unsatisfactory, owing to the limited clinical options for advanced-stage lesions [5]. Novel biomarkers and therapeutic targets are urgently needed in the near future.

Layilin (LAYN) is a transmembrane protein with a C-type lectin. Previous studies suggested that LAYN is involved in cancer cell invasion and could serve as a prognostic biomarker in human cancers [6–9]. A single-cell RNA sequencing analysis revealed that LAYN is upregulated on activated CD8^+^ T and Treg cells and represses the CD8^+^ T cell functions in vitro [10]. However, the underlying functions of LAYN and its interplay with immune cell infiltration in LIHC are still unclear.

To better understand the impacts of LAYN on LIHC development and the underlying mechanisms, we performed a comprehensive bioinformatics analyses in this study. Firstly, we evaluated the expression level and survival significance of LAYN in the LIHC cohort. The coexpressed genes and functional enrichment pathways were predicted. Next, the upstream noncoding RNAs (ncRNAs), including microRNAs (miRNAs), and long noncoding RNAs (lncRNAs) were investigated and analyzed. Then, the correlation between LAYN and immune cell infiltration in LIHC was explored. Our results provided novel insights into developing underlying prognostic biomarker and latent therapeutic target in LIHC.

## 2. Materials and Methods

### 2.1. UALCAN

UALCAN (http://ualcan.path.uab.edu/) is a public resource for the comprehensive analysis of gene expression data of 31 human cancer types from The Cancer Genome Atlas (TCGA) Project [11]. In this study, the “Expression Analysis” module was used to evaluate the mRNA level of LAYN across tumor and normal tissues and in different subgroups of patients with LIHC. The expression level and survival value of lncRNA HCG18 in LIHC were also determined using the UALCAN.

### 2.2. The Kaplan–Meier Plotter (KM Plotter)

The KM plotter (http://kmplot.com/) is a web platform to assess the effect of microarray-quantified genes (mRNA, miRNA, and protein) on survival in 21 human cancer types [12]. The prognostic values of LAYN and associated miRNAs in LIHC were obtained from the Kaplan–Meier plotter database. Patients were divided into a higher expression group and lower expression group by the best cutoff value.

### 2.3. LinkedOmics

LinkedOmics (http://www.linkedomics.org/) is a multidimensional dataset designed to analyze multiomics data for 32 TCGA Cancer types [13]. The miRNAs that are reversely associated with LAYN and the coexpressed genes of LAYN were screened via this website. In addition, the gene set enrichment analysis (GSEA) module of this website was used to predict the LAYN-related cellular processes and pathways.

### 2.4. GEPIA

The GEPIA database (http://gepia2.cancer-pku.cn/) is an interactive web portal that includes gene expression and prognostic data from TCGA and genotype-tissue expression (GTEx) projects [14]. The survival heatmaps of the top 50 genes with significant positive and negative correlations with LAYN are analyzed through this database.

### 2.5. UCSC Xena

UCSC Xena (http://xena.ucsc.edu/) includes multiomics and clinical data of human cancer and was used to evaluate the expression level of LAYN-correlated miRNAs [15].

### 2.6. StarBase

StarBase (https://starbase.sysu.edu.cn/) focuses on the RNA-RNA and protein-RNA interaction networks [16]. The miRNA-lncRNA and lncRNA-mRNA analysis modules were applied to perform correlation analyses for hsa-mir-148a-lncRNAs and LAYN-lncRNAs in LIHC. Moreover, the pancancer module was used to evaluate the expression level of lncRNA HCG18 in LIHC.

### 2.7. TIMER

TIMER (http://timer.cistrome.org/) contains the most comprehensive data on cancer immunity [17]. The correlation between LAYN expression and immune cell infiltration, as well as a variety of immune cell markers, was evaluated via the TIMER. The difference in 24 subtypes of immune cell infiltration between LAYN high and low groups was evaluated with “GSVA” package by ssGSEA algorithm.

### 2.8. Statistical Analysis

The plots and statistical results including either HR or *P* values in this study were obtained from the online databases mentioned above. The *P* value less than 0.05 (∗), 0.01 (∗∗), and 0.001 (∗∗∗) was considered as statistically significant. All data were originated from these public databases; our methods were performed in accordance with the relevant guidelines and regulations.

## 3. Results

### 3.1. LAYN Expression in Human Cancer

**Figure 1:**
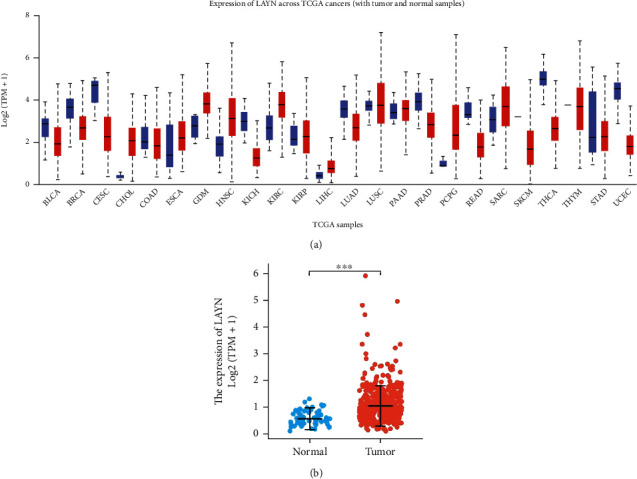
Pancancer analyses of LAYN expression in multiple types of human cancer (a) and the expression of LAYN in LIHC unpaired (b) and paired tissues (c).

To understand the potential role of LAYN in tumorigenesis, we first evaluated the expression level of LAYN in human cancers using the UALCAN database. As shown in Figure 1(a), LAYN is upregulated in several types of human cancer, including LIHC. The elevated mRNA expression of LAYN in LIHC tissues compared with the normal controls was further determined using TCGA data (Figures 1(b) and 1(c)).

### 3.2. Prognostic Significance of LAYN Expression and Its Correlation with Clinical Features in LIHC

**Figure 2:**
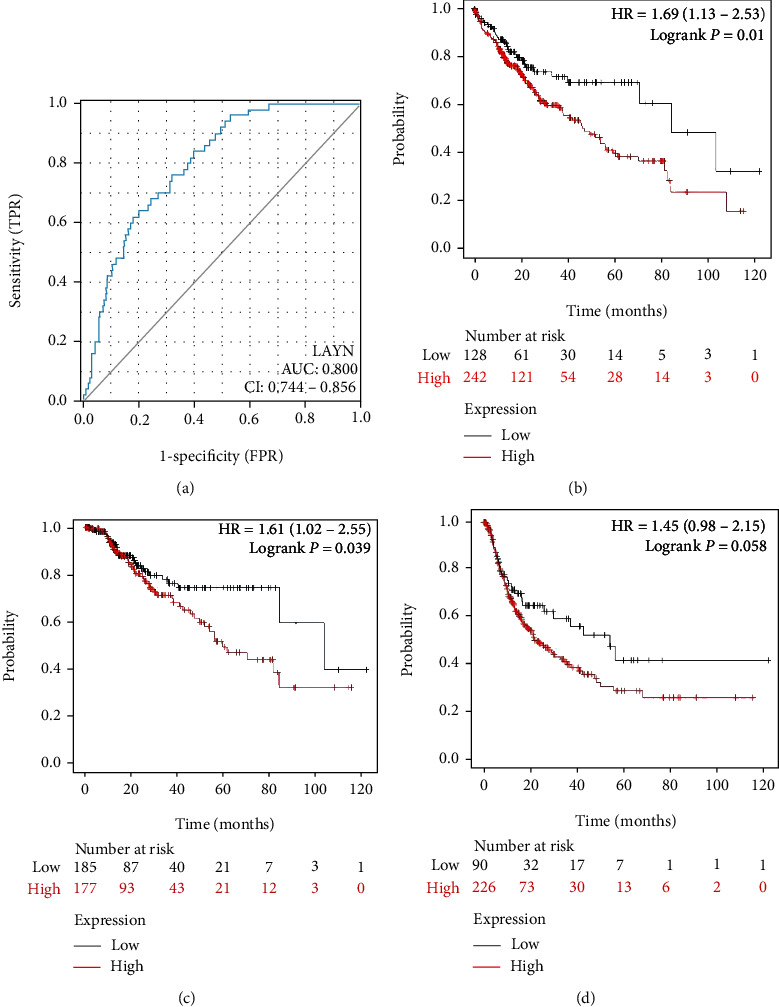
The prognostic value of LAYN in LIHC using the KM plotter database. (a) ROC curve of LAYN in LIHC. (b) OS: overall survival. (c) DSS: disease-specific survival. (d) RFS: recurrence-free survival.

**Figure 3:**
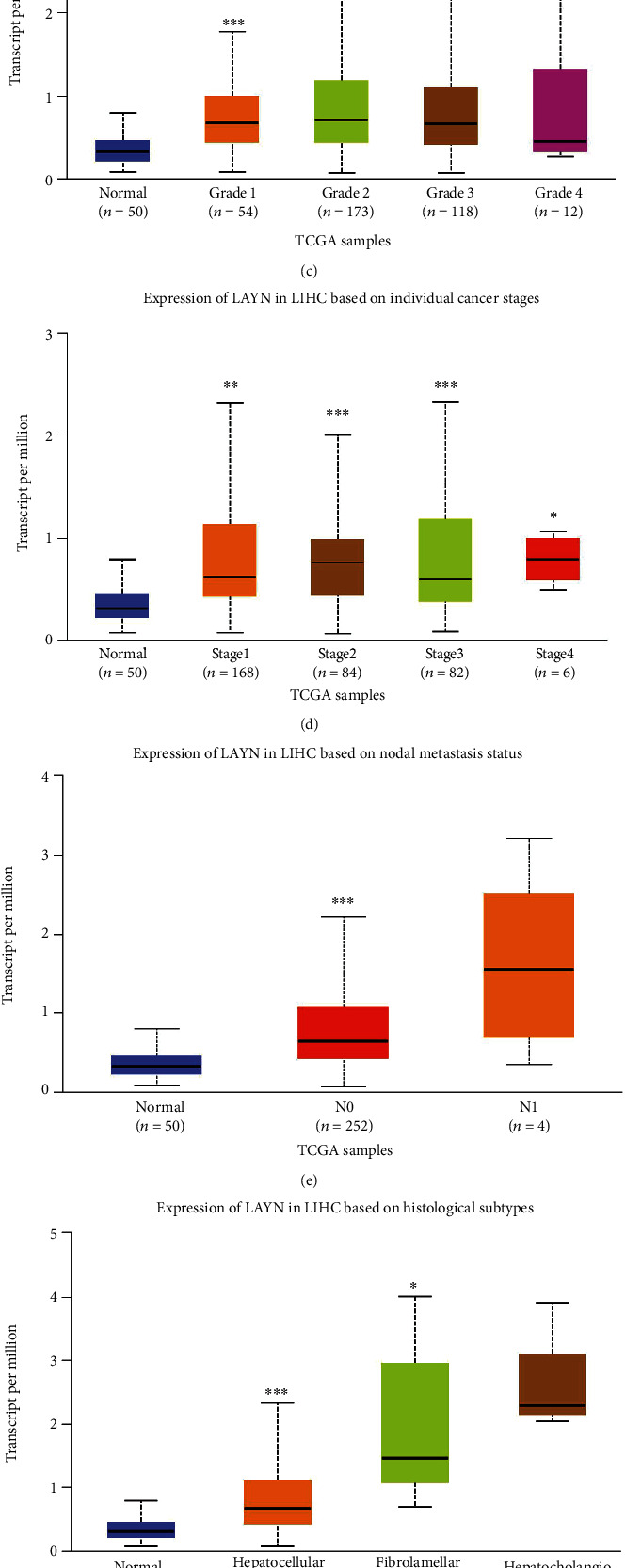
The correlation of LAYN with clinical features including age (a), gender (b), tumor grade (c), cancer stage (d), node metastasis (e), and histologic subtype (f). The nomogram (g) and calibration plot (h).

The ROC curve of LAYN distinguishing LIHC from healthy individuals is shown in Figure 2(a). The area under the curve (AUC) is 0.800 (95% CI: 0.744-0.856). Next, the prognostic value of LAYN expression in LIHC was explored using the Kaplan–Meier Plotter (KM Plotter) database. The inferior overall survival (OS), disease-specific survival (DSS), and recurrence-free survival (RFS) were observed in patients with high expression of LAYN (Figures 2(b)–2(d)). These results suggested that LAYN is overexpressed and might play a tumorigenic role in LIHC. In addition, we further performed a subgroup investigation and found the significantly higher expression of LAYN in the primary cancer patients than that of healthy controls in terms of age, gender, tumor grade, cancer stage, nodal metastasis status, and histological subtypes (Figures 3(a)–3(f)). A nomogram was designed for patients' survival prediction based on the LAYN expression and other clinical parameters including TNM stages, histologic grade, and age. The survival probabilities of 1 year, 3 years, and 5 years could be predictably calculated by the total points added for each variate in this model. The *C*-index is 0.646 (0.613-0.679) (Figure 3(g)), which shows good performance of the nomogram. Moreover, a calibration plot was generated. The plot indicates that the survival probabilities predicted by the nomogram are in good agreement with the observed survival probabilities (Figure 3(h)).

### 3.3. LAYN Coexpressed Genes and Functional Analyses

**Figure 4:**
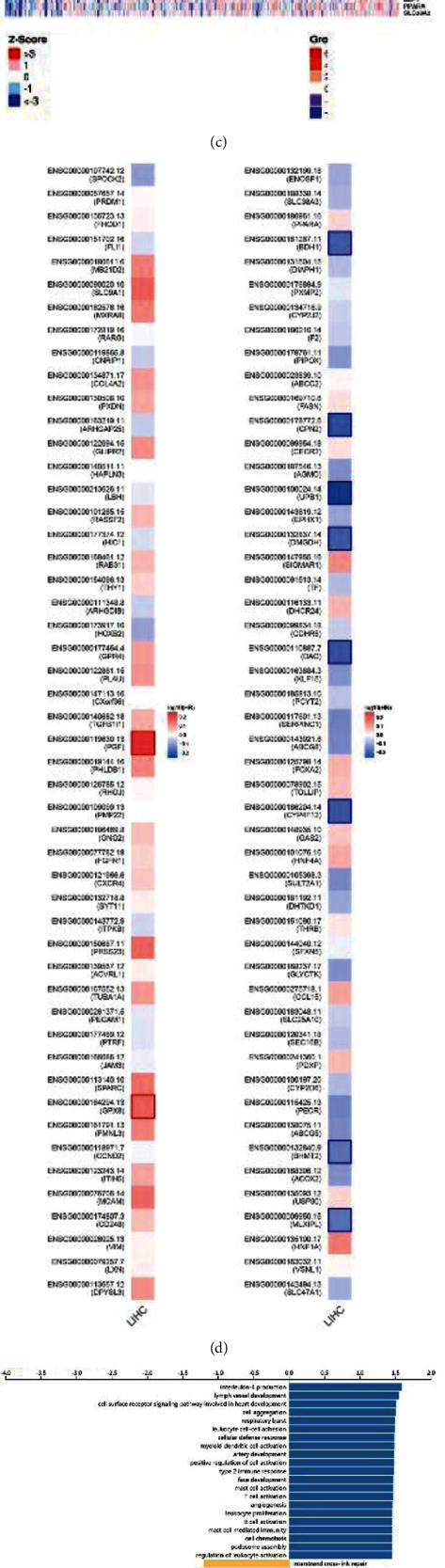
LAYN coexpressed genes and functional analyses in LIHC. (a) A scatter diagram of LAYN coexpressed genes. (b, c) The heatmaps of top 50 positively and negatively correlated genes of LAYN. (d) The survival maps of top 50 positively and negatively correlated genes of LAYN. (e, f) GO and KEGG analysis results of LAYN and its coexpressed genes in LIHC.

To further investigate the biological functions of LAYN in LIHC, we next explored the coexpressed genes of LAYN in the LIHC cohort. By Pearson correlation, genes that are positively and negatively associated with LAYN were labeled with red and green dots, respectively (FDR < 0.01) (Figure 4(a)). The top 50 genes with significant positive and negative correlations with LAYN were exhibited in heatmaps (Figures 4(b) and 4(c)). Then, the prognostic significance of these genes in the LIHC cohort was determined using the GEPIA database. As shown in Figure 4(d), 2 of the top 50 positively correlated genes are likely to be high-risk genes (*P* < 0.05), whereas eight of the top 50 negatively correlated genes are with low hazard ratio (HR) (*P* < 0.05). In addition, the gene set enrichment analysis (GSEA) module of LinkedOmics was used to gain insight of the involved biological processes and pathways of these genes. Gene Ontology (GO) term annotation showed that LAYN coexpressed genes mainly participate in cell adhesion and multiple inflammatory processes such as T cell activation, B cell activation, myeloid dendritic cell activation, mast cell activation, leukocyte proliferation and activation, cellular defense response, type 2 immune response, and interleukin-4 production (Figure 4(e)). Kyoto Encyclopedia of Genes and Genomes (KEGG) enrichment analysis revealed gene enrichment in biological pathways such as the T cell receptor signaling pathway; primary immunodeficiency; Th1, Th2, and Th17 cell differentiation; platelet activation; and inflammation-related disease such as asthma, allograft rejection, autoimmune thyroid disease, inflammatory bowel disease (IBD), and rheumatoid arthritis (Figure 4(f)). These data uncovered the widespread impact of LAYN on human immune response.

### 3.4. Predictive Analyses of Upstream miRNAs of LAYN in LIHC

**Figure 5:**
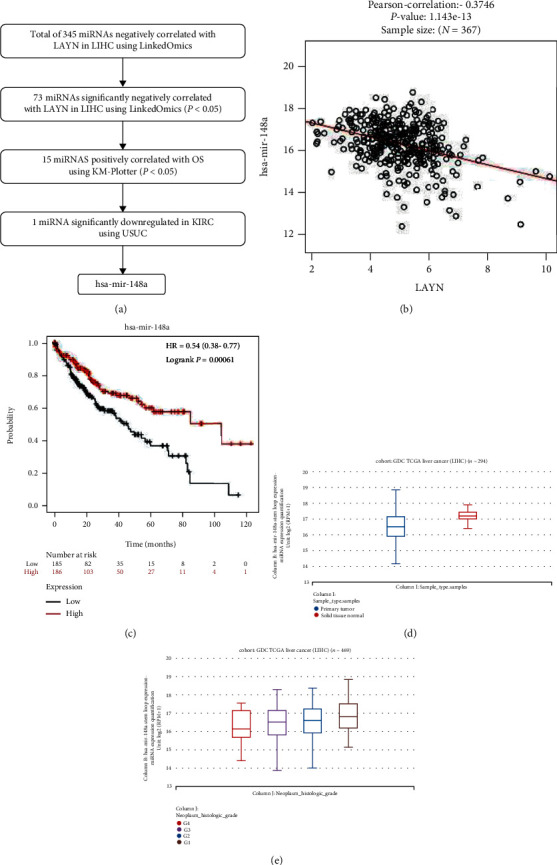
Prediction and analysis of upstream miRNAs of LAYN. (a) Analysis flow regarding correlations, gene expression, and survival. (b) The correlation between hsa-mir-148a and LAYN expression in LIHC. (c) The prognostic value of hsa-mir-148a in LIHC. (d, e) The expression of hsa-mir-148a in LIHC based on sample type (d) and tumor grade (e).

Past studies have changed our understanding of ncRNAs from “junk” transcripts to gene regulatory molecules. These ncRNAs, particularly miRNAs, lncRNAs, and circRNAs, could modify the expression of their target genes and have been identified as oncogenic drivers or suppressors in human cancers. According to the acknowledged ceRNA mechanism, lncRNAs typically regulate the specific mRNA expression at the posttranscriptional level by competitively targeting miRNAs [18]. In this study, we constructed a ceRNA network using comprehensive bioinformatics analyses. Firstly, we predicted the possible upstream miRNAs of LAYN in LIHC.  Figure 5(a) showed the analysis flow of screening miRNAs. As depicted, 73 out of 345 miRNAs are significantly negatively associated with LAYN expression using the LinkedOmics portal (*P* < 0.05). Among these miRNAs, 15 miRNAs positively correlate with OS of LIHC patients in the KM Plotter database, and only one miRNA of them, namely, hsa-mir-148a, was found downregulated in LIHC cancer tissues. Indeed, hsa-mir-148a is negatively correlated with LAYN expression (HR = −0.375, *P* < 0.001) (Figure 5(b)) and is associated with favorable survival outcome in LIHC. The expression level of hsa-mir-148a was determined in the UCSC Xena database, and the results showed that hsa-mir-148a is significantly downregulated in LIHC cancer tissues compared with the normal tissues (Figure 5(d)). In addition, the expression level of hsa-mir-148a was observed gradually decreased with the rise of tumor grade (Figure 5(e)). These data suggested that hsa-mir-148a might be the most likely upstream miRNA of LAYN in LIHC.

### 3.5. Predictive Analyses of Upstream lncRNAs of hsa-mir-148a in LIHC

**Figure 6:**
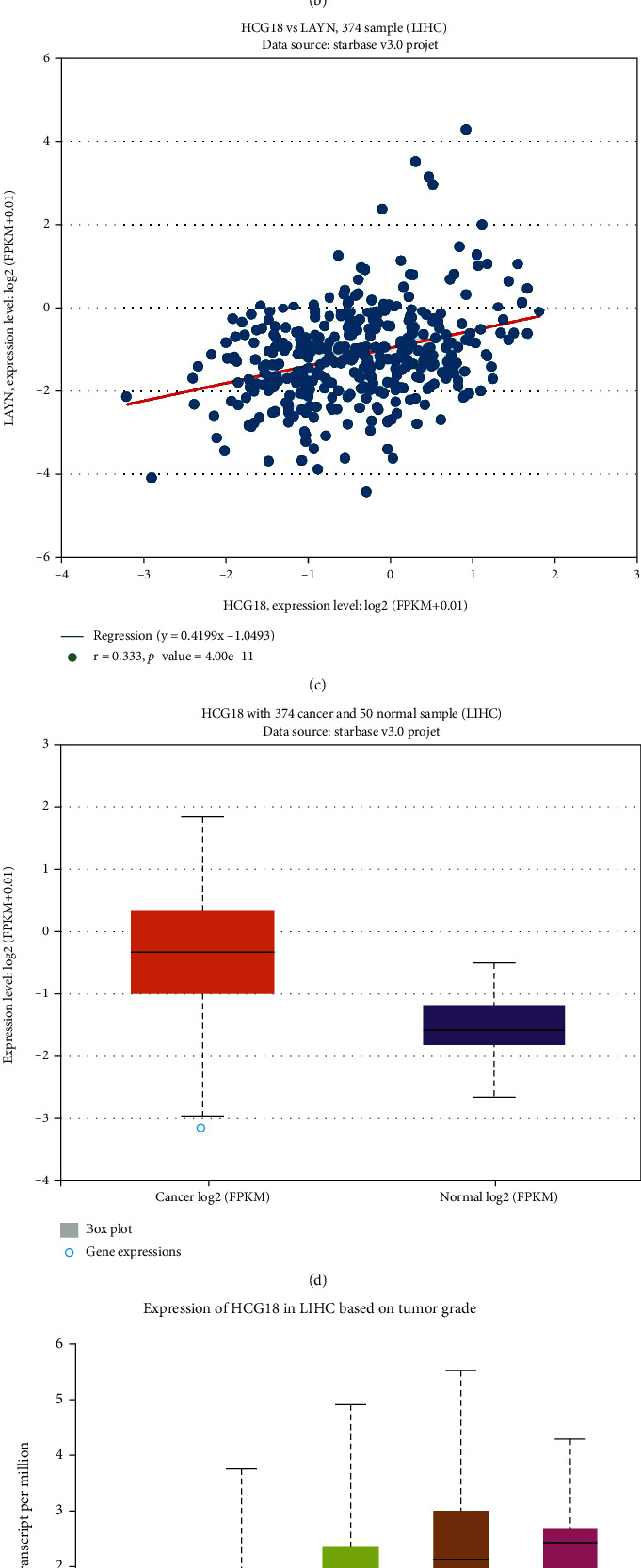
Prediction and analysis of upstream lncRNAs of hsa-mir-148a. (a) Analysis flow regarding correlations, gene expression, and survival. (b) The correlation between HCG18 and hsa-mir-148a expression in LIHC. (c) The correlation between HCG18 and LAYN expression in LIHC. (d, e) The expression of HCG18 in LIHC based on sample type (d) and tumor grade (e). (f) The prognostic value of HCG18 in LIHC.

miRNAs interact with other types of ncRNAs, such as circRNAs and lncRNAs, to regulate their biological properties. lncRNA could upregulate the target gene expression through competitively sequestrating mutual miRNAs. Therefore, the upstream lncRNAs should be negatively correlated with hsa-mir-148a expression, while they should be positively correlated with LAYN expression.  Figure 6(a) depicts the screening process of upstream lncRNAs. Using the StarBase database, a total of 14 lncRNAs were found to be significantly negatively correlated with hsa-mir-148a-3p, and 12 of them are positively correlated with LAYN expression. We next screened these lncRNAs by evaluating their expression levels in the LIHC cohort and found that 10 lncRNAs are upregulated in LIHC cancer tissues. Among them, only one lncRNA, namely, lncRNA HCG18, is negatively correlated with OS of LIHC patients using the UALCAN database. As shown in Figures 6(b) and 6(c), the expression of lncRNA HCG18 is negatively associated with hsa-mir-148a-3p (HR = −0.259, *P* < 0.001) and positively correlates with LAYN (HR = 0.333, *P* < 0.001). The expression of lncRNA HCG18 is elevated in LIHC cancer tissues (Figure 6(d)) and gradually increases in patients with higher tumor grades (Figure 6(e)). Moreover, the high expression of lncRNA HCG18 indicates inferior survival outcome of LIHC patients (Figure 6(f)). These data uncovered that lncRNA HCG18 might be the upstream regulatory molecule of the hsa-mir-148a-LAYN axis in LIHC.

### 3.6. Correlation between LAYN Expression and Immune Cell Infiltration in LIHC

**Figure 7:**
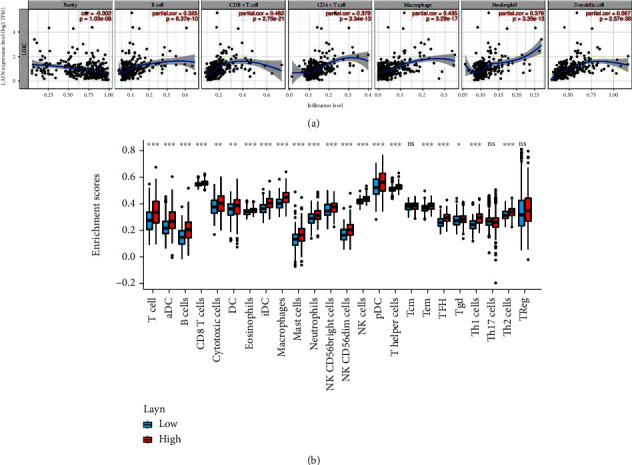
Correlation analyses between LAYN and six infiltrating immune cells (a) and 24 subtypes of tumor immune cells (b) in LIHC.

Of note, cancer cells are infiltrated by plentiful noncancer cells including immune cells, which are recruited in the tumor microenvironment (TME) and have vital impacts on cancer progression and survival [19, 20]. In view of the immune-related pathways which LAYN and its coexpressed genes are enriched in, we next investigated the correlation between LAYN and immune cell infiltration using the TIMER database. As shown in Figure 7(a), LAYN expression is significantly correlated with tumor purity (*R* = −0.302, *P* < 0.001) and the infiltration of six types of immune cells including B cells (*R* = 0.325, *P* < 0.001), CD4^+^ T cells (*R* = 0.379, *P* < 0.001), CD8^+^ T cells (*R* = 0.482, *P* < 0.001), macrophages (*R* = 0.435, *P* < 0.001), neutrophils (*R* = 0.379, *P* < 0.001), and dendritic cells (*R* = 0.567, *P* < 0.001) in LIHC tissues. Furthermore, the discrepant infiltration level between the high- and low-LAYN expression groups was observed in all 24 subtypes of immune cells apart from Tcm, Th17 cells, and Treg (Figure 7(b)).

We further explored the LAYN crosstalk with diverse tumor-infiltrating immune cell markers, including markers of B cells, CD4^+^ T cells, CD8^+^ T cells, tumor-associated macrophages (TAMs), monocytes, M1/M2 macrophages, neutrophils, DCs, and natural killer (NK) cells. Besides, other subpopulations of T cells, such as T helper 1 (Th1), T helper 2 (Th2), follicular helper T (Tfh), Th17, regulatory T (Tregs), and checkpoint marker expression, were also determined. After correlation adjustment by tumor purity, the results revealed that LAYN is significantly correlated with B cell biomarkers (CD19 and CD79A), CD8^+^ T cell biomarkers (CD8A and CD8B), CD4^+^ T cell biomarker (CD4), general T cell biomarkers (CD3D, CD3E, and CD2), monocyte biomarkers (CD86, C3AR1, CSF1R, CX3CR1, and CD14), TAM biomarker (CCL2, CD68, and IL10), M1 macrophage biomarkers (NOS2, IRF5, and PTGS2), M2 macrophage biomarkers (CD163, VSIG4, and MS4A4A), neutrophil biomarkers (ITGAM and CCR7), natural killer cell biomarkers (KIR2DL1, KIR2DL3, KIR2DL4, KIR3DL1, KIR3DL2, and KIR2DS4), and dendritic cell biomarkers (HLA-DPB1, HLA-DQB1, HLADRA, HLA-DPA1, CD1C, NRP1, and ITGAX) in LIHC (Table 1).

In addition, the expression of LAYN was significantly correlated with the expression of marker genes of different functional T cell subsets, including Th1 (TBX21, STAT4, STAT1, IFN-*γ*, and TNF-*α*), Th2 (GATA3, STAT6, and STAT5A), Tfh (BCL6), Th17 (STAT3 and IL17A), and Treg (FOXP3, CCR8, STAT5B, and TGF*β*) (Table 1).

**Figure 8:**
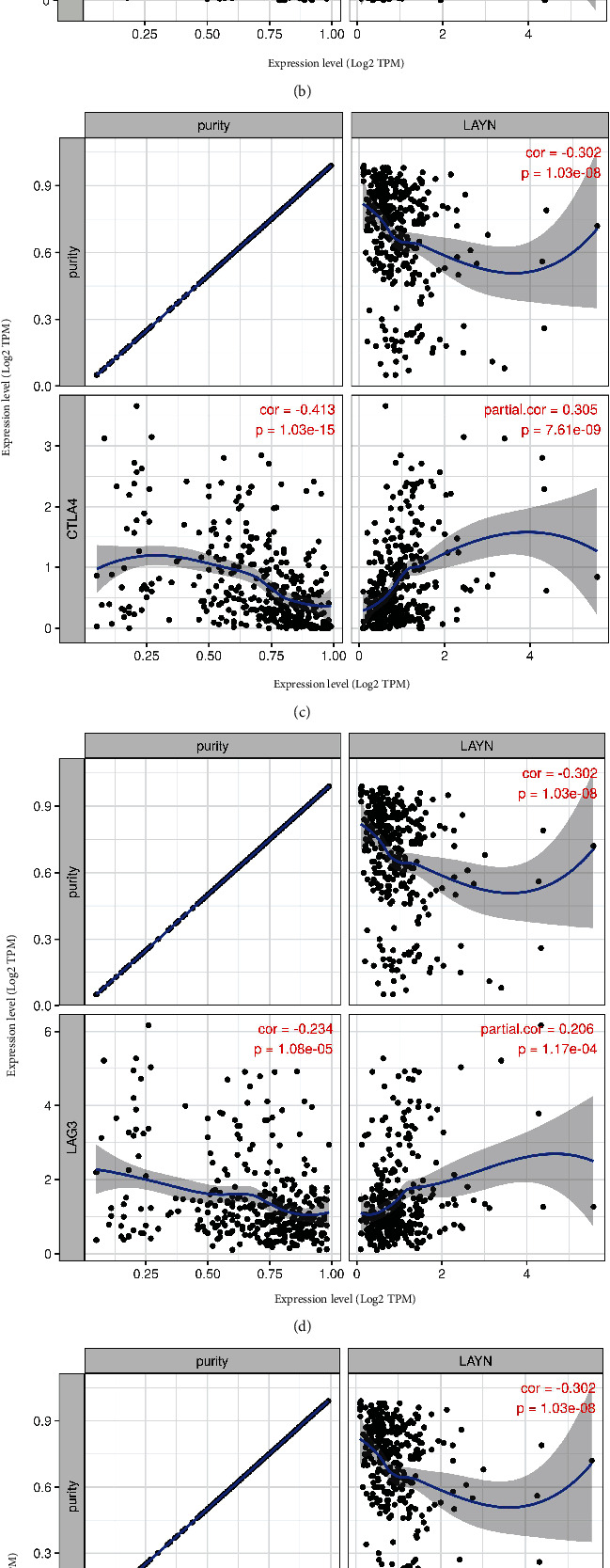
Correlation analyses between LAYN and immune checkpoint markers including PDCD1 (a), CD274 (b), CTLA4 (c), LAG3 (d), TIGIT (e), and TIM-3 (f).

Immune checkpoint inhibitors (ICIs), such as anti-PD-1, anti-PD-L1, and anti-CTLA-4 antibodies, have gained clinical efficiency in LIHC outcomes. Notably, LAYN expression is positively correlated with various immune checkpoint markers including PDCD1, CD274, CTLA4, LAG3, TIGIT, and TIM-3 (Table 1 and Figures 8(a)–8(f)). These results indicated that tumor immune cell infiltration is involved in LAYN-mediated tumorigenesis in LIHC.

## 4. Discussion

The global incidence and mortality of LIHC are still on the rise [21]. Due to the stealthiness of cancer progression and metastasis, the curative treatment for most patients remains a major challenge [22]. The liver is the largest immune organ in the human body; hepatic tumorigenesis is closely related to hepatocellular inflammation and fibrosis [23]. Indeed, LIHC is not only composed of cancer cells but also contains a large number of infiltrated immune cells, which have major impacts on cancer outcomes. Given the potential and delightful clinical efficiency of existing immunotherapeutic agents for advanced disease, novel immune biomarkers and treatment targets for LIHC are urgently needed in the near future.

LAYN, a transmembrane protein, mainly participates in cell adhesion. A recent study revealed that LAYN plays a role in T cell-related immunity. To better elucidate the potential functions and mechanism of LAYN in LIHC carcinogenesis, we performed this in silico analysis using multiple public databases to provide evidence for future studies.

In the present study, we found the elevated expression level of LAYN in the primary cancer patients than that of healthy controls regarding sample type, age, gender, tumor grade, cancer stage, nodal metastasis status, and histological subtypes. Next, the impacts of LAYN expression on the survival of LIHC patients were determined, showing that high expression of LAYN indicates poorer OS, DSS, and RFS of LIHC patients. Our data suggest that LAYN might play a protumorigenic role in LIHC, which was in accordance with the results of previous studies [7, 8].

To better understand the functions and involved biological processes of LAYN, we explored its coexpressed genes in LIHC (Figures 3(a)–3(c)). The survival maps of the top 50 positively and negatively correlated genes were analyzed via the GEPIA database (Figure 3(d)). Two positively correlated genes, namely, PGF and GPX8, were supposed as high-risk genes, while eight negatively correlated genes including BDH1, CPN2, UPB1, DMGDH, DAO, CYP4F12, BHMT2, and MLXIPL were deemed as low-risk genes. PGF is upregulated under hypoxic conditions and promotes cancer angiogenesis [24–26]. Previous studies indicated that overexpression of PGF is correlated with cancer progression and poorer prognosis of several types of cancer patients, including LIHC [27, 28]. In addition, GPX8, a member of the selenoproteome, is identified to promote cancer growth and progression in gastric cancer [29] and non-small-cell lung cancer [30], while the role of GPX8 in LIHC is not well identified.

For years, studies in tumor biology were confined to the expression and modification of the transcribed genomes, which only accounts for 2% of the entire human genome [31]. Over the past few decades, there has been increasing evidence that other types of RNAs, particularly ncRNAs, play vital roles in both normal cellular activity and human disease, including cancer progression [32, 33]. Noncoding RNAs are thought to act as cancer drivers or suppressors by regulating protein-coding gene expression. lncRNAs sponge miRNAs, thereby attenuating the inhibitory effect of miRNA on the downstream protein-coding target genes [34]. In this study, we further investigated the regulation mechanism of LAYN expression mediated by ncRNAs. Using multiple publicly available portals, we screened and supposed hsa-mir-148a as the most likely upstream miRNA of LAYN in LIHC. In line with our results, previous studies demonstrated that hsa-mir-148a is downregulated in LIHC cancer tissue and could suppress LIHC cell proliferation by regulating the MAPK pathway [35, 36]. Besides, plasma hsa-mir-148a expression level in LIHC patients is significantly lower than that in noncancer controls, suggesting that hsa-mir-148a might be a latent noninvasive biomarker for liver cancer screening [37].

On the basis of ceRNA mechanism, lncRNA could competitively sequestrate the mutual miRNAs to upregulate the target gene expression. We next explored the latent upstream lncRNAs, which are positively correlated with LAYN and negatively correlated with hsa-miR-148a in LIHC using the StarBase website. Our data revealed that lncRNA HCG18 played an oncogenic role and might be the upstream regulatory lncRNA of the hsa-mir-148a/LAYN axis in LIHC. Of note, lncRNA HCG18 was identified as an oncogene in several human cancers, including LIHC [38–40]. A recent study showed that lncRNA HCG18 is overexpressed in LIHC tissues and could promote the proliferation and migration of cancer cell lines [41]. These results are consistent with our data.

Moreover, GSEA revealed that LAYN participates in multiple inflammatory processes such as immune cell activation and the regulation of multiple subsets of T cell differentiation. These data were in line with the results of subsequent immune-related analyses, suggesting that the immunomodulatory property of LAYN might be responsible for its protumorigenic impact in LIHC.

Tumor lymphocyte infiltration is associated with cancer progression and survival. Our data uncovered the close association between LAYN expression and the vast majority of tumor-infiltrating immune cells as well as diverse immune cell markers. These results suggested that LAYN may regulate immune cell infiltration in the LIHC microenvironment. In addition, the expression of LAYN was also significantly correlated with the expression of marker genes of different functional T cell subsets, including Th1, Th2, Tfh, Th17, and Treg, indicating that LAYN might be involved in the regulation of T cell responses. Novel agents targeting checkpoint molecules such as PD1/PD-L1 and CTLA4 have gained early success in LIHC. Another finding of this study was the correlation between LAYN and several immune checkpoint markers in LIHC, suggesting that tumor immune escape might be involved in LAYN-mediated tumorigenesis in LIHC.

In conclusion, the present study provided a perspective on the oncogenic roles of LAYN via regulating tumor immune cell infiltration in LIHC. The upstream regulatory mechanism of the HCG18/hsa-mir-148a/LAYN axis was also identified. Future studies should focus on the underlying molecular mechanisms of LAYN on TIICs, which may further broaden the immunotherapy options for LIHC.

## Figures and Tables

**Table 1:** Correlation analyses between LAYN and markers of immune cells in LIHC via the TIMER database.

		LIHC			
None		Purity	
Description	Gene markers	Cor	*P*	Cor	*P*

CD8^+^ T cell	CD8A	0.42	∗∗∗	0.35	∗∗∗
	CD8B	0.37	∗∗∗	0.291	∗∗∗

CD4^+^ T cell	CD4	0.418	∗∗∗	0.356	∗∗∗

T cell (general)	CD3D	0.396	∗∗∗	0.315	∗∗∗
	CD3E	0.471	∗∗∗	0.391	∗∗∗
	CD2	0.447	∗∗∗	0.364	∗∗∗

B cell	CD19	0.341	∗∗∗	0.255	∗∗∗
	CD79A	0.395	∗∗∗	0.306	∗∗∗

Monocyte	CD86	0.55	∗∗∗	0.495	∗∗∗
	C3AR1	0.541	∗∗∗	0.481	∗∗∗
	CD115 (CSF1R)	0.523	∗∗∗	0.449	∗∗∗
	CX3CR1	0.435	∗∗∗	0.395	∗∗∗
	CD14	-0.098	0.06	-0.132	∗

TAM	CCL2	0.502	∗∗∗	0.408	∗∗∗
	CD68	0.368	∗∗∗	0.282	∗∗∗
	IL10	0.439	∗∗∗	0.354	∗∗∗

M1 macrophage	INOS (NOS2)	0.238	∗∗∗	0.224	∗∗∗
	IRF5	0.292	∗∗∗	0.309	∗∗∗
	COX2 (PTGS2)	0.51	∗∗∗	0.429	∗∗∗

M2 macrophage	CD163	0.433	∗∗∗	0.357	∗∗∗
	VSIG4	0.4	∗∗∗	0.341	∗∗∗
	MS4A4A	0.437	∗∗∗	0.398	∗∗∗

Neutrophils	CD66b (CEACAM8)	-0.007	0.899	-0.036	0.509
	CD11b (ITGAM)	0.396	∗∗∗	0.319	∗∗∗
	CCR7	0.448	∗∗∗	0.35	∗∗∗

Natural killer cell	KIR2DL1	0.132	∗	0.106	∗
	KIR2DL3	0.271	∗∗∗	0.225	∗∗∗
	KIR2DL4	0.284	∗∗∗	0.25	∗∗∗
	KIR3DL1	0.266	∗∗∗	0.247	∗∗∗
	KIR3DL2	0.236	∗∗∗	0.2	∗∗∗
	KIR3DL3	0.068	0.194	0.067	0.215
	KIR2DS4	0.162	∗∗∗	0.167	∗∗∗

Dendritic cell	HLA-DPB1	0.535	∗∗∗	0.454	∗∗∗
	HLA-DQB1	0.421	∗∗∗	0.342	∗∗∗
	HLA-DRA	0. 512	∗∗∗	0.433	∗∗∗
	HLA-DPA1	0.545	∗∗∗	0.468	∗∗∗
	BDCA-1 (CD1C)	0.383	∗∗∗	0.289	∗∗∗
	BDCA-4 (NRP1)	0.535	∗∗∗	0.5	∗∗∗
	CD11c (ITGAX)	0.482	∗∗∗	0.416	∗∗∗

Th1	T-bet (TBX21)	0.402	∗∗∗	0.316	∗∗∗
	STAT4	0.323	∗∗∗	0.27	∗∗∗
	STAT1	0.396	∗∗∗	0.386	∗∗∗
	IFN-*γ* (IFNG)	0.296	∗∗∗	0.221	∗∗∗
	TNF-*α* (TNF)	0.379	∗∗∗	0.283	∗∗∗

Th2	GATA3	0.534	∗∗∗	0.467	∗∗∗
	STAT6	0.244	∗∗∗	0.255	∗∗∗
	STAT5A	0.483	∗∗∗	0.435	∗∗∗
	IL13	0.098	0.058	0.062	0.254

Tfh	BCL6	0.206	∗∗∗	0.224	∗∗∗
	IL21	0.095	0.066	0.072	0.18

Th17	STAT3	0.368	∗∗∗	0.338	∗∗∗
	IL17A	0.122	∗	0.113	∗

Treg	FOXP3	0.271	∗∗∗	0.219	∗∗∗
	CCR8	0.448	∗∗∗	0.397	∗∗∗
	STAT5B	0.264	∗∗∗	0.342	∗∗∗
	TGF*β* (TGFB1)	0.541	∗∗∗	0.467	∗∗∗

Checkpoints	PDCD1	0.424	∗∗∗	0.36	∗∗∗
	CD274	0.402	∗∗∗	0.366	∗∗∗
	CTLA4	0.382	∗∗∗	0.305	∗∗∗
	LAG3	0.253	∗∗∗	0.206	∗∗∗
	TIGIT	0.466	∗∗∗	0.408	∗∗∗
	TIM-3 (HAVCR2)	0.534	∗∗∗	0.48	∗∗∗

## Data Availability

All results in the current study were generated from the publicly available databases described above and available from the corresponding author upon reasonable request.
